# CT-guided microwave ablation combined with thoracoscopic resection for multiple ground-glass nodules: a single-session treatment strategy

**DOI:** 10.3389/fmed.2026.1791023

**Published:** 2026-04-28

**Authors:** Danyang Wu, Yunxiao Zhou, Hongqiang Sun, Bao Wang, Jingzhen He, Bo Liu

**Affiliations:** 1 Department of Radiology, Qilu Hospital of Shandong University, Jinan, China; 2 Department of Radiology, Yangxin County People's Hospital, Binzhou, China

**Keywords:** ground-glass nodule, hybrid therapy, microwave ablation, multiple pulmonary nodules, uniportal video-assisted thoracoscopic surgery

## Abstract

**Purpose:**

To explore the safety, feasibility and clinical value of CT-guided microwave ablation (MWA) combined with immediate uniportal video-assisted thoracoscopic surgery (Uni-VATS) in the treatment of multiple pulmonary ground-glass nodules (GGNs).

**Methods:**

The clinical, radiographic, surgical, and pathological data of patients with multiple GGNs who underwent CT-guided MWA combined with Uni-VATS from October 2022 to December 2024 were retrospectively reviewed.

**Results:**

A total of 31 patients (7 males, 24 females; mean age 59.3 ± 9.5 years) with multiple GGNs underwent CT-guided MWA combined with Uni-VATS. Among these patients, 78 lesions were treated: 32 with MWA and 46 with surgical resection. The mean nodule size of the ablated lesions was 7.4 mm, while the mean nodule size of the resected lesions was 12.2 mm. Postoperative complications occurred in one patient (pneumothorax and subcutaneous emphysema), which resolved with symptomatic treatment before discharge. The technical success rate and clinical efficacy of CT-guided microwave ablation were 100%, with no severe complications or procedure-related mortality. During follow-up, no local recurrence or metastatic progression was observed.

**Conclusion:**

Our preliminary experience suggests that a single-session, combined approach of CT-guided MWA immediately followed by Uni-VATS for the treatment of multiple GGNs is safe and feasible, and may provide an alternative treatment method for more patients, especially those who cannot tolerate the simultaneous removal of multiple nodules.

## Introduction

Lung cancer is one of the malignant tumors with the highest morbidity and mortality in the world (1–3). With the increasing health needs of people and the widespread application of high-resolution computed tomography (HRCT), the detection rate of lung nodules by chest imaging has been significantly improved. These nodules are mostly ground glass opacities (GGO) (4). Two or more GGOs synchronously found in the same lobe, lung, or bilateral lungs, with multifocal origins rather than intrapulmonary metastasis, are multiple pulmonary ground-glass nodules (GGNs) (5). Based on pathological characteristics, multiple GGNs can be further divided into subtypes such as atypical adenomatous hyperplasia (AAH), adenocarcinoma *in situ* (AIS), microinvasive lung adenocarcinoma (MIA), and invasive adenocarcinoma (IA) (6).

Surgical resection, especially by video-assisted thoracoscopic surgery (VATS), is considered the preferred treatment for multiple GGNs, but it is not suitable for patients with poor physical condition (7). In addition, to completely remove all nodules, complex multi-segmental resection, bilobectomy, or even bilateral surgery or pneumonectomy may be required, which not only increases the intraoperative risk and postoperative complication rate, but may also seriously affect the patient’s quality of life due to impaired cardiopulmonary function. Percutaneous image-guided thermal ablation (IGTA) is a precise and minimally invasive treatment option for thoracic tumors, sparing the lung parenchyma (8). It is increasingly recognized as a safe and effective local treatment alternative to surgical resection and radiation therapy. These thermal ablation techniques offer multiple advantages over surgery, including shorter recovery time, less bleeding, lower infection rates, and fewer major complications. Radiofrequency ablation (RFA), microwave ablation (MWA), cryoablation, and laser ablation represent the primary IGTA techniques. Among these, MWA offers distinct advantages, including higher energy delivery, shorter procedure duration, and reduced susceptibility to the heat sink effect. Moreover, accumulating clinical evidence has demonstrated both the efficacy and safety of MWA in treating early-stage primary lung cancers (9–12).

The management of multiple GGNs demands individualized strategies, considering each nodule’s risk, location, follow-up changes, and patient factors (13). An optimal approach must effectively treat lesions while maximizing lung function preservation. The ablation combined with surgical resection treatment model has been successfully applied to multiple liver metastases, and its experience can provide a reference for the treatment of multiple pulmonary GGNs (14–16). Previous studies have confirmed that surgical resection combined with ablation is a safe and effective treatment for patients with multiple high-risk GGNs, especially those who have underwent pneumonectomy or have poor lung condition and are in urgent need of preserving lung function (17–19). However, in the traditional treatment model, ablation and surgical resection are usually performed in stages, with long intervals, and patients need multiple anesthesia and imaging evaluations.

In this study, we propose a single-session, combined approach in which CT-guided MWA is immediately followed by Uni-VATS. This preliminary study aims to evaluate the procedural feasibility, safety, and early clinical efficacy of this integrated approach and provide new ideas for the minimally invasive treatment of multiple GGNs.

## Patients and methods

### Patient selection

This retrospective study was approved by the Qilu Hospital of Shandong University Research Ethics Committee (KYLL-2025(ZM)-1338). Due to the retrospective nature of the study, informed consent was not required. This study retrospectively collected the clinicopathological data of patients who underwent CT-guided MWA immediately followed by Uni-VATS between October 2022 and December 2024. The inclusion criteria were as follows: (1) age ≥ 18 years; Eastern Cooperative Oncology Group (ECOG) performance status score of 2 points; (2) chest CT confirmed ≥ 2 GGOs (pGGO or mGGO), and all ground-glass nodules were found before the start of treatment or during follow-up; and (3) Following CT-guided MWA and localization, patients underwent immediate surgical resection, which included wedge resection, segmentectomy, or lobectomy. The exclusion criteria were as follows: (1) a history of myocardial infarction or stroke within 6 months before surgery; (2) severe bleeding tendency, platelet count <50*10^9/L, or prothrombin time >18 s or prothrombin time activity <40%; (3) patients were admitted to the hospital for other treatments (radioactive particle implantation, radiotherapy, etc.) at the same time or at different times; and (4) patients were not followed up or died.

The therapeutic strategy for each nodule was formulated through multidisciplinary team (MDT) discussion, adhering to the principles of maximal lung preservation and oncological radicality. The decision-making framework integrated nodule characteristics, patient factors, and current guideline recommendations.

MWA was preferred for nodules with the following features: (1) Smaller size (typically ≤ 10 mm in diameter); (2) Deep location within the pulmonary parenchyma, where wedge resection would necessitate removal of an excessive volume of normal lung tissue or might be technically challenging; and (3) Predominantly pure ground-glass opacity (pGGO) on CT, suggesting a likely pre-invasive or minimally invasive pathology that is amenable to local control with ablation.

Uni-VATS was preferred for nodules with the following features: (1) Larger size (typically > 10 mm) or with a significant solid component (mGGO); (2) Peripheral location amenable to wedge resection with adequate margins; (3) Nodules showing interval growth or increasing solidity on serial CT scans; and (4) The need for definitive pathological diagnosis and staging to guide further management.

The decision to intervene in subcentimeter GGNs was reserved for nodules exhibiting high-risk features, aligning with a selective, proactive management strategy. These features included: (1) multifocality, indicative of an elevated adenocarcinoma risk and allowing combined diagnostic-therapeutic intervention within a field cancerization context; (2) documented radiological progression, defined as growth ≥2 mm or increased solid component size/density on serial CT; (3) presence of suspicious morphological features (e.g., lobulation, spiculation, pleural retraction) despite small size; and (4) high patient anxiety with a strong preference for definitive treatment after thorough counseling, provided the multidisciplinary team deemed the procedural risk acceptable.

The following data were collected for analysis: patient and nodule characteristics, including MWA parameters, medical glue positioning parameters, surgical treatment data, complications, and procedural characteristics of local efficacy. The primary endpoints were the safety and feasibility of this combined technique.

### CT-guided MWA

MWA was performed with ECO-100C MWA in the CT-intervention room for persistent highly suspicious malignant nodules and biopsy-proven malignant lesions. The microwave transmitting frequency was 2,450 50 MHz with an output power of 0–150 W. The effective length of the microwave antenna was 130–180 mm with an outer diameter of 15G-18G, and a water-circulating cooling system was used to reduce the surface temperature of the antenna. CT was used for guiding the MWA and for evaluating the results.

Preoperatively, the patient’s position (supine, lateral, prone, etc.) was determined by observing the CT images, the body surface marking line was posted, the level and location of the lesion were determined based on the CT scan, and the path of puncture needle entry was developed. The procedure was performed aseptically and under local anesthesia at the puncture site. The ablation needle was gradually pierced into the lesion under CT guidance, and a cable was used to connect the ablation needle with the microwave ablator and the cold circulatory tube. The ablation time and power were determined according to lesion size, lesion changes, and patient tolerance.

### Preoperative localization

Following ablation, medical glue localization of nodules requiring surgical resection was performed in the CT-intervention room. Under CT guidance, an 18-G Chiba puncture needle (China) was precisely inserted toward the target nodules. The CT scanner was then rescanned to confirm accurate positioning at the expected marking point. After confirming proper placement, the core of the Chiba puncture needle was withdrawn, and medical glue (Italy) was injected at a controlled rate of 0.15–0.2 mL using a 1-mL syringe. To ensure complete delivery of the glue, 0.1 mL of 5% glucose solution was subsequently injected to flush any residual medical glue from the needle into the lung tissue. The needle was maintained in position for 3–5 s before rapid removal. The puncture site was then dressed. Following confirmation of the patient’s stable condition in the CT-intervention room, the patient was transferred to the operating room for Uni-VATS surgical treatment.

### Surgical resection

Following MWA and medical glue localization procedures, patients were immediately transferred to the operating room for general anesthesia and surgical resection. All ablation and surgically treated pulmonary nodules included in the study were located in the ipsilateral lung. All patients underwent preoperative physical examination, hematological examination, electrocardiogram, pulmonary function tests, chest CT plain, and contraindications to surgery were excluded. The surgical procedure was prepared by multiple specialists and informed to the family. General anesthesia was administered to all patients, and the patient’s vital signs were monitored during the operation. The patient’s position was determined according to the location of the lesion, a thoracoscope was inserted to dissect the free artery, vein, and bronchus and close and cut them off, resect the lesion and the part of the lung lobe where it was located, clear the mediastinal lymph nodes of the lung hilum, and put in a chest drain to flush the chest cavity. The surgical specimen was sent for pathologic examination, and the patient returned to the ward after being awake.

### Follow-up

All patients were followed up after surgery. Follow-up was done in the outpatient clinic or by telephone. Chest CT, tumor markers, and abdominal ultrasound were reviewed every 3 months in the first year after surgery; every 6 months in the second year after surgery, and annually thereafter. Ablation site progression, distant metastasis, and tumor death were monitored by routine chest CT. If any recurrence signs or symptoms were detected, further evaluation including CT, MRI, and positron emission tomography (PET) was performed. This study reports the early-term (6-month) safety and procedural feasibility outcomes. All patients are enrolled in an ongoing long-term follow-up program. According to our follow-up protocol, surveillance is continued with chest CT and tumor marker assessment every 6 months in the second year post-procedure and annually thereafter to monitor for local recurrence, new primary lesions, and distant metastasis. Future analyses will focus on long-term oncological efficacy, including local control rates and overall survival.

### Statistical analysis

Data are expressed as median (range) for continuous variables and as the total number (percentage) for categorical variables. The statistical analysis was performed using SPSS version 27.

## Results

### Patient and nodule characteristics

A total of 31 patients with multiple GGNs who underwent simultaneous CT-guided MWA combined with Uni-VATS were included. Among them, there were 7 men and 24 women, with a mean age of 59.3 ± 9.5 years. Thirteen patients had underlying diseases, including 6 with diabetes, 1 with chronic obstructive pulmonary disease (COPD), and 6 with hypertension. The detailed description of the patient and lesion characteristics is provided in Table 1.

**Table 1:** Patient characteristics.

Variables	Number/mean ± SD
Gender	
Male	7 (22.6%)
Female	24 (77.4)
Age (years)	59.3 ± 9.5
Tabaco	
Smoking	4 (13%)
Non-smoking	27 (87%)
Comorbidities	
Yes	13 (41.9%)
No	18 (58.1%)
Family history	
Yes	3 (10%)
No	28 (90%)
Maximum nodule diameter (mm)	11.8 ± 5.1

### Ablation and localization details

**Figure 1:**
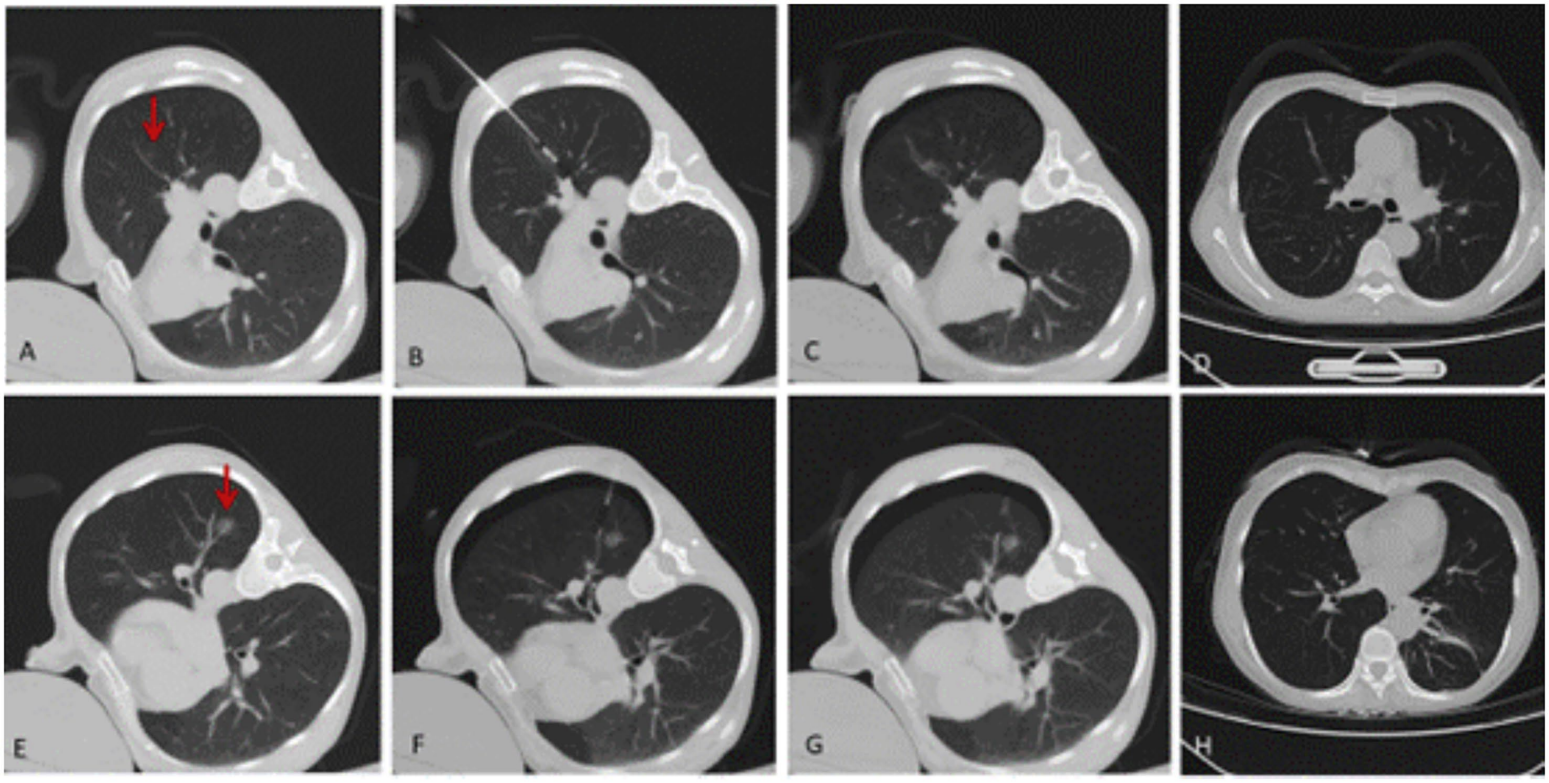
A 52-year-old female with bilateral multiple pulmonary nodules showing no regression after 6-month follow-up with anti-inflammatory therapy. The small GGO in the left upper lobe was treated with microwave ablation, and the large GGO in the left lower lobe was localized with medical glue and then surgically resected. CT images showing **(A)** left upper lobe GGO (arrow); **(B)** CT-guided microwave ablation needle placement for left upper lobe GGO; **(C)** immediate post-ablation CT showing minimal pneumothorax (arrowhead); **(D)** 2-year follow-up CT demonstrating ablation scar without recurrence; **(E)** left lower lobe mixed GGO with vascular penetration (arrow); **(F)** medical glue localization needle tip positioning; **(G)** post-localization CT showing glue deposition (star); **(H)** 2-year follow-up CT after wedge resection showing clear surgical margin without recurrence. Final pathology confirmed minimally invasive adenocarcinoma in the resected specimen.

All target nodules were successfully treated with CT-guided MWA and medical glue localization (technical success rate 100%). No major procedure-related complications were observed. Minor adverse events included bleeding in two cases (6%) and transient cough in twelve cases (38%), all of which resolved with symptomatic management. Fluoroscopic imaging intraoperatively confirmed clear visualization of all ablation zones, with no instances requiring supplemental localization techniques. The specific ablation and positioning information is shown in Table 2. Figure 1 illustrates a representative case of a 54-year-old female patient with bilateral multiple GGNs who successfully underwent the combined procedure.

**Table 2:** Procedure characteristics of CT guided MWA and preoperative localization.

Variables	Number/mean ± SD
Power of MWA (W)	36.7 ± 6.7
Time of MWA (min)	2.9 ± 0.2
Location of nodules treated using MWA	
Right upper lobe	10 (31%)
Right middle lobe	6 (19%)
Right lower lobe	5 (16%)
Left upper lobe	7 (22%)
Left lower lobe	4 (12%)
Size of nodules treated using MWA (mm)	7.4 ± 3.4
Ablation success rate	31 (100%)
Localization time (min)	14.8 ± 3.2
Localization distance (mm)	13.1 ± 3.1
Localization-surgery interval (min)	108.9 ± 52.5
Localization complications	
Pneumothorax	0
Bleeding	2 (6%)
Irritable cough	12 (38%)

### Surgical outcomes

A total of 46 nodules were surgically resected. The specific nodule locations and surgical methods for each patient are shown in Table 3. The average surgical time was 84.7 ± 30.3 min, and the surgical time was extended to 180 min for one elderly patient with poor general condition. It is worth noting that complete resection of the lesion was achieved in all cases, with an R0 resection rate of 100%. The perioperative results and complications are presented in Table 4.

**Table 3:** Characteristics of nodules undergoing Uni-VATS approach.

Variables	Number/mean ± SD
Surgical procedures	
Wedge resection	2 (6.4%)
Segmentectomy	14 (45.2%)
Combined segmentectomy	5 (16.1%)
Lobectomy	10 (32.3%)
Total number of nodules	46
Right upper lobe	16 (34.8%)
Right middle lobe	7 (15.2%)
Right lower lobe	8 (17.4%)
Left upper lobe	4 (8.7%)
Left lower lobe	11 (23.9%)
Size of nodules treated using Uni-VATS (mm)	12.2 ± 4.3
Postoperative pathology	
AAH	5 (11%)
AIS	12 (26%)
MIA	16 (35%)
IA	13 (28%)

**Table 4:** Summary of perioperative outcomes.

Variables	Number/mean ± SD
Total of operation time (min)	84.7 ± 30.3
Intraoperative blood loss (ml)	48.6 ± 18.2
Postoperative chest tube duration (day)	3.2 ± 0.5
Length of hospital stay	10.6 ± 3.2
Hospital mortality rate	0
Complications	
Pneumothorax	1 (3%)
Pneumonia	0
Hemoptysis	0
Subcutaneous emphysema	0

### Complication profile

The single-session, combined approach used in this study showed excellent safety performance. Only one patient developed mild postoperative pneumothorax, who recovered smoothly after symptomatic treatment, and no other serious complications or perioperative deaths occurred. The 6-month follow-up results showed that all patients survived and no signs of local recurrence were found, which preliminarily confirmed the short-term safety and effectiveness of this procedure. The follow-up time needs to be extended to evaluate the long-term efficacy.

## Discussion

In this initial series, the combination of CT-guided MWA and Uni-VATS is a feasible approach for managing multiple GGNs, with favorable safety and survival outcomes. The data from this study demonstrated that all 31 patients treated with this strategy successfully completed the procedure, with all targeted nodules ablated as planned and no severe treatment-related complications observed.

These findings indicate that the combined MWA and surgical approach is a safe and feasible method for managing GGNs, which is consistent with the results reported by Wang et al. (20). Zhang et al. demonstrated that the combination of CT-guided RFA and VATS is a safe and feasible therapeutic approach for patients with multiple pulmonary nodules (21). In contrast, our study employed MWA, a promising and relatively novel ablation technique (22). Compared with RFA, MWA can reach higher ablation temperatures in a shorter time and is less affected by high impedance caused by high temperature and heat sink effect, thus forming a larger and more uniform ablation zone. The ablation range mainly depends on the power output and action time of MWA (23, 24). Moreover, MWA offers distinct advantages for treating gas-rich lesions like ground-glass nodule lung cancers, as it can directly ablate these targets without requiring saline injection between the electrode and lesion. This capability is particularly valuable given the frequent recurrence risk in lung cancer patients, where maximal lung preservation is critical. MWA’s tissue-sparing properties enable multiple treatments without significant functional compromise. Additionally, MWA precisely targets small, deeply located nodules that are often non-palpable and challenging to completely excise via conventional wedge resection.

Zeng et al. demonstrated that electromagnetic navigation bronchoscopy (ENB)-guided MWA combined with surgical resection offers favorable short-term safety and efficacy in the treatment of MPN (25). In contrast, the CT-guided MWA combined with ablation adopted in the present study exhibits distinct technical features and clinical advantages. The ENB technique, being performed via natural airways, is associated with a theoretically lower risk of pneumothorax and bleeding, making it particularly suitable for centrally located nodules or those adjacent to major vessels (26). However, ENB is limited by its high equipment cost, technical complexity, and dependence on favorable bronchial anatomy (27). In comparison, CT-guided MWA offers several benefits: first, it has a broader application range unrestricted by bronchial accessibility, especially for peripheral small nodules; second, it provides high positioning accuracy with real-time imaging ensuring precise placement of the ablation needle and localization markers; and third, it significantly reduces both equipment and operational costs, facilitating wider adoption of the technique.

Harrison et al. explored a hybrid technique that combines percutaneous ablation and wire-assisted wedge resection (iCART) for minimally invasive treatment of multiple pulmonary metastases (28). This study reported four cases treated with iCART, demonstrating that this combined therapy provides minimally invasive and comprehensive personalized treatment for patients with multiple pulmonary metastases. This technique uses cone beam CT (ARTIS Pheno system) to guide ablation and resection in a hybrid operating room at the same time, aiming to achieve lesion removal while maximally preserving lung tissue. The results of the study showed that iCART achieved personalized treatment through a single anesthesia and avoided cross-departmental referral. However, the construction and operation costs of hybrid operating rooms are high, and they have not yet been widely popularized in clinical practice. In this study, we present a single-session, combined treatment strategy: first, CT-guided lesion ablation and localization are completed in the radiology department (the routine configuration of most medical institutions), and then the patient is immediately transferred to the operating room for local lesion resection.

The single-session, combined approach employed in this study demonstrated excellent safety profiles, with all 31 patients successfully completing both CT-guided MWA and surgical resection without experiencing any severe procedure-related complications. The 6-month follow-up results showed that all patients survived and no signs of local recurrence were found, which preliminarily confirmed the short-term safety and effectiveness of this procedure. This result shows that the immediate combination of CT-guided MWA with video-assisted thoracoscopic surgery represents a safe and feasible innovative approach for managing pulmonary GGNs. Despite not utilizing expensive hybrid operating room equipment, this study successfully achieved safe and precise combined management of multiple nodules in a conventional operating room setting through optimized workflow. This combined treatment strategy warrants widespread clinical adoption as a valuable alternative to hybrid operating room techniques, particularly for patients with multiple GGNs requiring lung function preservation. While not conducted in a single physical hybrid room, our sequentially combined protocol achieves the primary clinical objective of a “one-stop” strategy: to resolve multiple lesions in a single, coordinated hospital encounter, thereby avoiding the delays, repeated anesthesia, and logistical burdens of staged procedures. This represents a pragmatic and accessible implementation of the integrated treatment concept.

The medical glue localization technology used in this study has significant clinical application value. The main component of medical glue is cyanoacrylate, which is a material with good biocompatibility. It can quickly polymerize and solidify after contacting with tissue fluid (29, 30). This property enables clear marker formation when injected around target nodules under CT guidance, facilitating intraoperative identification and improving surgical efficiency. Compared with alternative techniques, medical glue localization offers distinct advantages: (1) cost-effectiveness and procedural simplicity; and (2) an extended time window between localization and surgery. According to the report of Wang et al. (31), the surgery can be completed within 48 h after positioning. The data of this study showed that the average positioning time was 14.8 ± 3.2 min, and the average interval from positioning to surgery was 108.9 ± 52.5 min. The mean operative time was 84.7 ± 30.3 min, with preoperative precise localization improving surgical efficiency. Safety outcomes demonstrated no severe complications among the 31 localized patients. Minor bleeding occurred in 2 cases (6%), successfully managed conservatively. Twelve patients (38%) experienced transient cough that resolved spontaneously. The medical glue’s rapid solidification effectively sealed puncture tracts, minimizing pneumothorax and bleeding risks. While the glue’s irritant properties may induce coughing (particularly with deep lung injections), optimized technique and sedation mitigated these effects without compromising procedural accuracy or surgical outcomes. Future studies should optimize injection parameters for different nodule locations to enhance precision and minimize adverse events.

This study adopted a novel multimodal treatment strategy. The results showed that this sequential combined treatment regimen was safe and effective, and no local recurrence was observed in short-term follow-up. Although this hybrid surgery shows good application prospects, its long-term efficacy still needs further research and confirmation. Notably, radical surgery remains the standard treatment for early-stage lung cancer. MWA exhibits limitations for peripheral nodules near major vessels or bronchi, with increased risks of postoperative pain, pleural effusion, and pneumothorax. Furthermore, the long-term outcomes of MWA for early lung cancer remain debated. Conversely, complete surgical resection may compromise pulmonary function, quality of life, and future treatment options. Following MDT evaluation and shared decision making (SDM), we implemented this hybrid strategy to optimize both therapeutic efficacy and quality of life. In summary, treatment decisions for patients with GGOs should be individualized through MDT discussions to achieve the best balance between treatment effect and quality of life.

Our study has some limitations. First, this is a retrospective, single-center study, which inevitably has some inherent defects. Therefore, our results need to be verified by prospective studies. Second, all ablated nodules were persistent lesions with high clinical suspicion of malignancy, and a subset received preoperative biopsy confirmation. However, not all lesions underwent definitive pathological verification. Third, this study focused on the evaluation of CT-guided MWA positioning technology, and has not yet systematically compared the differences in the combined use of other emerging navigation technologies (such as electromagnetic navigation bronchoscope or hybrid operating room fluorescent markers) with MWA.

## Conclusion

In summary, this study demonstrates the initial application of a combined strategy integrating CT-guided MWA with immediate Uni-VATS for patients with multiple ground-glass nodules. This combined protocol was successfully implemented in our cohort with a high technical success rate and a favorable short-term safety profile, providing a practical workflow for the precise management of multifocal lesions in a single session. This integrated treatment protocol merits widespread clinical adoption as a significant alternative to hybrid operating room techniques, particularly for patients with multiple GGNs requiring pulmonary function preservation.

## Data Availability

The original contributions presented in the study are included in the article/supplementary material, further inquiries can be directed to the corresponding author.
